# The Economic Value of the Deciduous Teeth

**Published:** 1912-06-15

**Authors:** M. Evangeline Jordon


### THE ECONOMIC VALUE OF THE DECIDUOUS TEETH.

BY M. EVANGELINE JORDON, D.D.S.

The environment of the American people has entirely changed within the lifetime of one generation, and the connection between the environment and the teeth has not yet forced itself upon the minds of the public. A perfect dental equipment is one of the best gifts to mankind, and environment is one of the great destroyers or preservers of the dental equipment.

This was recognized when a parallel was drawn between the perfect denture of Sitting Bull, who had lived the free life of the plains and had eaten the simple primitive food, and the broken carious teeth of his grandson, who had suffered from the conditions of civilization.

Our change of environment has been slow, but that it is just as fatal is shown by school examinations in different cities, where the number of children needing dental care runs from 75 to as high as 97 per cent.

The value of the teeth with regards to the State—that is, the effect upon the health of society at large and upon the taxes they must pay—is but little realized by the profession, and it is not even imagined by the laity.

In his last report Dr. Ebersole, the chairman of the National Committee on Oral Hygiene, tells us that when the mouths of the school children are put into a healthy condition they can do 20 per cent more work. The lack of such work, he estimates, is an annual loss to the taxpayers of the city of Cleveland of half a million dollars.

Cleveland is one city in the United States, and conditions are similar in all communities.

This is only one way in which neglected teeth may increase taxes. The cost of caring for the young criminals might be greatly lessened by keeping the mouths of poor children in a healthy condition. We should then have fewer young criminals, because workers in juvenile courts find carious teeth one of the predisposing causes of viciousness and delinquency. Often these children become honest and upright when their mouths are made healthy. A step farther and the cost of maintaining prisons, courts and penitentiaries would be lessened if there were fewer criminals growing up to fill them.

Hospitals are a great expense. Those who work in clinics for tuberculous children tell us that such children always have carious teeth. Go into any hospital and examine the mouths of the inmates and you will be satisfied that if their teeth had been kept in repair many of them would not need to be there.

Another heavy item of expense to the taxpayer is in maintaining asylums for the insane, which each year are being more crowded. Some of these unhappy people would be well and self-supporting if their teeth had been cared for, but now they are a tax upon the people.

And last, but saddest of all, when old age is reached many people must be cared for by the State because they were unsuccessful in life. One fifth, or more, of their strength was lost by neglected teeth.

This is needless waste, and is largely due to the fact that people think that because the deciduous or baby teeth are to be shed that they need no care. Nothing was ever farther from the truth. These teeth are needed for use between the ages of two and twelve, and under our present state of civilization every dollar spent in keeping the mouth in perfect health during this period brings better returns in health and strength than three dollars later on.

It was recognized very early in the study of the causes for carious teeth that the child who was raised at the mother's breast had better teeth, better shaped jaws, and was probably freer from adenoids and enlarged tonsils, than the bottle-fed baby.

It remained for dentists practicing exclusively for children to discover the very serious results that may be traced to bottle feeding.

The first of these is the early decay of the teeth before the second year. This may usually be traced to the lactic acid action upon the upper incisors of the children who have been fed upon bottle food that is too sweet, such as condensed milk, goat's milk, etc.

In these cases a stain appears upon the teeth during the last part of the first year, and in a few months these stained areas deepen into cavities, often causing the teeth to be broken down to the gums by the middle of the third year.

If the child has care, the abscess which follows the growth of the cavity and death of the pulp may be treated and the teeth filled and restored to usefulness.

My records show many such cases of children ranging from eighteen months to two and one-half years of age.

Each of these children needed besides such treatments several small fillings in other teeth, which if neglected would have gone through the same destructive stages of inflammation of the bacteria-invaded pulp, its death and suppuration, and later alveolar abscess, followed by a necrosed area of the alveolar process surrounding the root.

Possibly the busy physicians have overlooked these apparently little trifles without realizing how prevalent and how serious are the dead pulps in children's teeth.

An abscess upon the finger is a serious thing, but how much more serious it would be considered if its discharges were all carried into the system.

Where there is one tooth with an abscess another will soon be in the same condition, because mastication upon the approximal and occluding teeth becomes difficult and painful, and the destructive bacteria burrow toward the pulps of these teeth with less disturbance from the process of mastication.

The blood is laden with pus germs absorbed directly by the tissues surrounding the roots of the teeth and also by the way of the stomach and intestines, because the slightest pressure upon the tooth squeezes great drops of creamy pus into the food being prepared for digestion.

Each tooth with an abscess reduces the resisting power of the child, until when there are five or six, or even seven, as one of my little patients of three and one-half years had, great quantities of pus are absorbed daily and very little resistance is made against the poisoning.

Many a little grave, yes thousands of little graves, hide the victims of septicemia, although the child appeared to succumb to some simple ailment.

The little patient suffering with seven abscesses was brought from a neighboring town, and referred to me because the dentists who had examined her found her extreme irritability a hindrance in doing satisfactory work for her relief. In six weeks her teeth were filled, but for several months pus would reappear at some point of the necrosed areas about the roots. These were all finally healed, and at a recent visit, after a year's absence, her gums were perfectly healthy and her teeth all in service.

A year and a half ago she passed through a serious run of typhoid fever where her physicians say she could not have escaped death had her mouth not been in a perfectly healthy condition.

Generally, conditions of this sort are brought to the attention of the physicians first, and if they do not recognize them the blame should rest at their doors.

Some do recognize the danger from the pus, and extract the teeth, without realizing the injury they may be doing to the proper occlusion of the permanent set.

Never extract a deciduous tooth except for its immediate successor, is an axiom in dentistry, which should prevent the early sacrifice of such teeth as might easily be restored to health and usefulness by a few simple treatments.

The prolonged use of the nursing bottle causes the upper arch to grow high and narrow, which results in a permanent lengthening of the face and malocclusion of the arches. The upper front teeth may project and prevent the closing of the mouth. In such cases the child may breathe through the mouth, and is then subject to inflammation of throat and tonsils. The air passages of the nose become smaller and growth of adenoids is induced.

If the upper teeth are broken off very early the lower jaw, having no support, may sag forward and remain in the protruding position.

Where artificial feeding cannot be avoided the watchfulness of the mother may do much in prevention of these troubles. The nose must be kept clean so that there is no obstruction to free breathing. The bottle must be taken from the child as soon as empty and "*pacifiers*" must never be used. The mouth must be kept very clean, and as soon as the teeth appear they must be kept free from stain. If the food is sweet, magnesia helps to counteract the acid and to keep the stomach more healthy.

The deciduous teeth are for use during the time of greatest development of the child, and the shortest lived of these, the incisors, should last for six years. The molars which are replaced by the bicuspids should be in use for eight to ten years, and any interference with the usefulness of these teeth interferes with the nutrition and the growth of the child. It may not always show in the physical appearance but it always interferes with the nervous system. Children whose teeth have been badly neglected are frequently the victims of a serious breakdown, which often becomes most apparent as they approach puberty.

Dentistry, like education, should be begun in childhood. If prophylactic work is begun before any stains appear upon the teeth and is carried along without interruption there is every reason to believe that there never will be even a roughening of the enamel of a single tooth. The exception to this rule is where the child is a victim of severe malnutrition due to some extreme febrile disorder, as the result of scarlet fever, diphtheria, measles, etc., or syphilis, in which case the growth of the teeth may be stopped during the development of the enamel and result in atrophied teeth, those mis-shapen, stunted teeth so difficult to preserve and so much less useful because of the small surface of occlusion.

The first permanent molar is most often the victim of atrophy and may generally be traced to such a disturbance occuring between birth and the third year.

The preservation of the first permanent molar is one of the great problems in dentistry. Erupting in the sixth year, it is generally mistaken by the laity for a deciduous tooth. When the mouth is full of caries this tooth often begins to decay before it is fully erupted.

When caries reach the pulp before the tenth year it is almost certain to be lost, as the roots are not completely formed until nearly four years after eruption.

One of the greatest mistakes made is to think that this most valuable tooth of the second denture can be permanently filled before puberty. I can safely say that fully as many teeth are lost as saved when filled with silver amalgam in childhood. Prior to puberty we frequently find an acid saliva depositing the destructive coating of mucous upon the teeth, similar to the conditions during the early months of pregnancy. Then the bacteria penetrate between the wall of the filling and the tooth and protected by the filling, develop great colonies which undermine the tooth and penetrate the pulp, while externally there is no sign until the whole tooth suddenly falls to pieces like the collapse of a building with a weak foundation.

The teeth, like the forest and rivers of the nation, are one of our greatest natural resources, and should be understood and conserved with equal care, as much of the health and happiness of the nation depend upon their usefulness.

Their conservation is one of the simplest and easiest matters, when faithfully continued from babyhood to adult life.

Then all fear of dental work is unknown, because if, as the result of an illness, some small cavities do form they are filled before they become sensitive.

Where prophylactic work is practiced children not only lose all fear of the dentist but look forward to their monthly appointments as a pleasant form of entertainment.

Prophylactic work being done once a month, a constant supervision is kept of the oral hygiene practiced at home, and any mistakes in the use or lack of use of the brush can be corected.

Many pregnant women are allowed to suffer with their teeth when the dental work necessary for their relief would be far less injurious to the development of the child than the sleepless nights of pain which quickly sap a woman's strength.

The poisoning from abscessed teeth or pus pockets about the necks of the teeth very seriously hampers the proper development of the child, and such conditions have been instrumental in causing premature delivery.

Prophylactic work for women during pregnancy, when begun in the earlier months is doing much to stop the rapid caries common during that time and to prevent the incipient pyorrhea alveolaris to which later the mouth of the mother so often falls a victim.

If the fear and the pain of dentistry can be relegated to the past with other plagues and horrors another step upward will be taken in the progress of science and eugenics.—*Pacific Gazette.*

